# Evaluation of uric acid levels, thyroid function, and anthropometric parameters in Japanese children with Down syndrome

**DOI:** 10.3164/jcbn.17-55

**Published:** 2017-08-18

**Authors:** Tomomi Niegawa, Kimitaka Takitani, Ryuzo Takaya, Manabu Ishiro, Yuichi Kuroyanagi, Keisuke Okasora, Yukako Minami, Takuya Matsuda, Hiroshi Tamai

**Affiliations:** 1 Department of Pediatrics, Osaka Medical College, 2-7 Daigakumachi, Takatsuki, Osaka 569-8686, Japan; 2 Department of Pediatrics, Saiseikai Ibaraki Hospital, 2-1-45 Mitsukeyama, Ibaraki, Osaka 567-0035, Japan; 3 Department of Pediatrics, Saiseikai Suita Hospital, 1-2 Kawazonocho, Suita, Osaka 564-0013, Japan; 4 Department of Pediatrics, Hirakata City Hospital, 2-14-1 Kinyahonmachi, Hirakata, Osaka 573-1013, Japan

**Keywords:** Down syndrome, uric acid, thyroid function, anthropometry

## Abstract

Down syndrome, caused by trisomy 21, is characterized by congenital abnormalities as well as mental retardation. From the neonatal stage through adolescence, patients with Down syndrome often have several complications. Thus, it is important to attain knowledge of the prevalence of these comorbidities in children with Down syndrome. We, therefore, evaluated the biochemical data, thyroid function, and anthropometric parameters, and analyzed the association among them in Japanese children and early adolescents with Down syndrome. There was no difference in the prevalence of obesity and overweight between boys and girls. The level of uric acid was higher in boys than in girls. Moreover, the prevalence of hyperuricemia was also higher in boys than in girls (approximately 32% and 10%, respectively). The prevalence of subclinical hypothyroidism in children with Down syndrome was approximately 20%, with no significant sex differences. The levels of uric acid and dehydroepiandrosterone-sulfate were positively associated with age, while the levels of thyroid-stimulating hormone and free thyroxine had a negative association with age. Overall, children with Down syndrome, exhibit a higher incidence of hyperuricemia. Therefore, uric acid levels, as well as thyroid function, from childhood to early adulthood should be monitored in this patient cohort.

## Introduction

Trisomy 21, which is responsible for Down syndrome, is the most common aneuploidy chromosomal abnormality and affects approximately 1 in 800 infants in the United States.^(1,2)^ Down syndrome is characterized by congenital abnormality as well as mental retardation, with its distinctive dysmorphic features that lead to recognition of trisomy 21 at birth. Newborn infants with Down syndrome have often several complications, including congenital heart defects, gastrointestinal manifestations, hearing loss, hematological abnormalities, ophthalmological disorders, congenital hypothyroidism and hypotonia.^(3)^ It is important that the medical problems occurring in infants with Down syndrome are promptly evaluated, with the institution of appropriate treatments during this period. The degree of developmental retardation due to Down syndrome varies widely during childhood; however early, optimal intervention can enable the acquisition of appropriate developmental skills.^(1)^ During infancy, respiratory tract infection occurs frequently, with an increase in the incidence of obstructive sleeping apnea and seizures.^(3)^ Throughout childhood, growth and weight gain are routinely monitored, and developmental social skills should be evaluated. Children presenting autism, attention-deficit/hyperactivity disorder, or behavioral or psychiatric problems should be referred to medical professionals.^(3)^ Adolescents with Down syndrome develop behavioral and psychiatric problems, and physicians should monitor the development of several conditions in this patient cohort, including atlantoaxial instability, leukemia, diabetes, obstructive sleep apnea, arthritis and seizures.^(4)^

Uric acid is the final product of purine metabolism in humans and higher primates. Clinical observations have demonstrated the close association between elevated levels of serum uric acid and the development of hypertension among children and adults.^(5)^ Hyperuricemia, which is the primary risk factor for developing symptomatic gout, is robustly associated with the development of cardiovascular diseases and metabolic disorders, including coronary artery disease, hypertension, renal failure, metabolic syndrome, obesity and type 2 diabetes.^(6,7)^ The prevalence of hyperuricemia in children has increased concomitant with the higher incidence of obesity, especially in developed countries,^(8)^ which can be attributed to increased fructose intake from sweetened foods and beverages.^(9)^ Following the report on elevation of the level of serum uric acid in children and adults with Down syndrome, more than half a century ago,^(10)^ there have been several clinical studies that have focused on this area of research.^(11,12)^

Thyroid hormones play a critical role in growth, development of tissues, cellular differentiation and cellular metabolism, especially the maintenance of energy homeostasis.^(13,14)^ The physiological function of thyroid hormones can be exerted via genomic action (i.e., by its activation of thyroid hormone receptors), or non-genomic actions, including its effects on integrins, signal transducing proteins, and direct actions on mitochondria.^(13)^ From the neonatal stage through adolescence, the occurrence of hypothyroidism is higher than that of hyperthyroidism.^(15)^ During neonatal screening, congenital hypothyroidism is found more frequently in patients with Down syndrome, compared with the expected frequency in the general population.^(16,17)^ Moreover, hyperthyroidism, and more exceedingly hypothyroidism, occurs frequently in patients with Down syndrome, from childhood through adulthood.^(18)^ The American Academy of Pediatrics recommends the measurement of thyroid-stimulating hormone (TSH) levels, annually, in children with Down syndrome to monitor thyroid function through early adulthood.^(3)^

The prevalence of obesity and being overweight in children and adolescents has risen during the past decades, eliciting critical concerns about the future of public health.^(19)^ Approximately a quarter of all children are either obese or overweight.^(20)^ In addition to higher-income nations, the incidence rate of being overweight and obesity in children has also increased in lower- and middle-income nations during the past three decades.^(21)^ The development of obesity is closely related to several factors, including the living environment, nutrition and genetics.^(22)^ The elevation in the total calorie intake and reduction in physical activity during childhood and adolescence have accelerated adiposity. Furthermore, genetic disorders including Down syndrome, Prader-Willi syndrome and Bardet-Biedl syndrome, are known risk factors of childhood obesity. The American Academy of Pediatrics Expert Committee reported the recommendations for staged treatment and management program of obese and overweight children.^(23)^ A systematic review demonstrated that concentrated and multifaceted treatment intervention was most effective against adiposity during early childhood.^(24)^ Metabolic syndrome, which is defined as abdominal obesity, hypertension, dyslipidemia, and hyperglycemia, is observed in approximately a quarter of obese children.^(20,25)^ Metabolic syndrome in childhood is associated with a critical risk for the development of type 2 diabetes, hypertension, dyslipidemia, and cardiovascular disease in adulthood, which is closely related to morbidity and mortality of adults.^(26)^

Given the abovementioned comorbidities that children with Down syndrome can develop during adulthood, medical professionals, including pediatricians and physicians, should closely supervise this patient population. Thus, the knowledge of the prevalence of these comorbidities in children with Down syndrome is crucial. Currently, however, there is a lack of information about the prevalence of these comorbidities among Asian children with Down syndrome. In the present study, therefore, we evaluated the association between biochemical data, thyroid function, and anthropometric parameters among Japanese children and early adolescents with Down syndrome.

## Materials and Methods

### Study participants

We enrolled a total of 102 children with Down syndrome in the current study, including 62 boys and 40 girls, aged 5–15 years, from January 2013 to November 2014. The children attended the clinic for Down syndrome in the Department of Pediatrics, Osaka Medical College Hospital, in the morning, 2–3 h after breakfast, with no exertion during the previous day. Blood samples were collected and blood pressure and anthropometric parameters, including height and body weight, were measured. We excluded children treated with thyroid or cardiac medication. This study was approved by the ethical committee of Osaka Medical College (code number 1385) and informed consent was obtained from the parents of all the participants.

### Measurement of anthropometric data and blood pressure

Children wore light clothes and were barefoot when their heights and body weights were measured. Blood pressure was measured in the right arm using an automated sphygmomanometer. In the present study, children who were overweight and obese were defined has having a body mass index [BMI; body weight (kg)/height (m^2^)] of ≥85th and <95th percentiles, and ≥95th percentile, respectively, based on their age and sex.^(27)^ The height standard deviation (SD) was calculated by dividing it with the value at the 50th percentile for age and sex, based on the national statistics for Japanese children by the Ministry of Health Labor and Welfare.^(28)^

### Biochemical data

Once the blood samples were collected, the serum levels of total-cholesterol, alanine aminotransferase (ALT), and uric acid were measured using an AU 5800 automated analyzer (Beckman Coulter, Brea, CA). The level of high-density lipoprotein cholesterol (HDL-C) was assayed using an enzymatic color method, while the level of non-high-density lipoprotein cholesterol (HDL-C) was calculated by subtracting the level of HDL-C from total cholesterol. Levels of TSH, free thyroxine (fT4), and free triiodothyronine (fT3) were analyzed using an electrochemiluminescence immunoassay (ECLIA). The reference ranges used for TSH, fT4, and fT3 were 0.5–5.0 µIU/ml, 0.9–1.7 ng/dl, and 2.3–4.3 pg/ml, respectively. Additionally, thyroid-stimulating antibody (TSAb), anti-TSH receptor antibody (TRAb), and anti-thyroid peroxidase antibody (TPOAb) were assessed using enzyme immunoassay, radio receptor assay, and ECLIA, respectively. The normal reference ranges used for TSAb, TRAb, and TPOAb were <180%, <1.0 IU/L, and <16 IU/ml, respectively. Subclinical hypothyroidism was defined as TSH >5.0 µIU/ml. Dehydroepiandrosterone sulfate (DHEA-S) was quantified using a chemiluminescence enzyme immunoassay. Hyperuricemia was defined as a serum uric acid level of more than 6.0 mg/dl among participants of both sexes and all ages, in keeping with previous reports.^(29,30)^

### Statistical analysis

Results are expressed as mean ± SD. Sex differences for several parameters were calculated using unpaired *t* tests. Simple linear regression coefficients and multiple regression analysis were used to examine the correlation among each parameter, in children and early adolescents with Down syndrome, using JMP 9 software (SAS Institute Inc., Cary, NC). The prevalence of complications, including obesity and overweight, hyperuricemia, subclinical hypothyroidism, and positive anti-thyroid antibodies in children with Down syndrome was analyzed using chi-square tests. Statistical significance was set as *p*<0.05.

## Results

### Study cohort characteristics

The anthropometric parameters and the characteristics of patients with Down syndrome are shown in Table 1. Anthropometric parameters, including height, weight, height-SD, and BMI percentile did not significantly differ between boys and girls. The serum levels of both uric acid and fT4 were significantly higher in boys than in girls. Other biochemical parameters, however, were not significantly different between boys and girls.

### Prevalence of complications in patients with Down syndrome

**Fig. 1:**
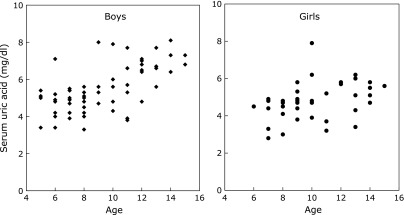
Scatter dot plot of the level of uric acid, in relation to age, in boys and girls with Down syndrome.

The prevalence of obesity and overweight, hyperuricemia, and subclinical hypothyroidism in all patients with Down syndrome was 14.7, 23.5, and 19.6%, respectively (Table 2). The incidence rate of obesity or being overweight was not different between boys and girls. A total of 32.3% of boys and 10% of girls with Down syndrome presented hyperuricemia, and the prevalence of hyperuricemia was significantly higher in boys than in girls. The distribution of uric acid levels in relation to age, among boys and girls with Down syndrome, is shown in Fig. 1. The prevalence of subclinical hypothyroidism in boys and girls was 17.7 and 22.5%, respectively, and there were no sex differences. Moreover, we examined thyroid antibodies in 55 boys and 36 girls. The prevalence of positive thyroid antibodies, including TSAb, TRAb, and TPOAb, was 13.2% (i.e., 5.5 and 25%, in boys and girls, respectively). TSAb, TRAb and TPOAb were detected in 5.5, 7.7, and 8.8% of individuals with Down syndrome, respectively (i.e., antibodies were detected in 1.8, 1.8, and 1.8% of boys, respectively, and in 11.1, 16.7, and 11.1% of girls, respectively). The prevalence of positive thyroid antibodies in girls was markedly higher than that in boys.

### Correlation among uric acid levels, thyroid hormones, and dehydroepiandrosterone-sulfate levels

We analyzed the correlation among several parameters, including uric acid levels, thyroid function, non-HDL-C levels, DHEA-S levels, and age using a simple linear regression analysis (Table 3). Uric acid levels were significantly associated with age and DHEA-S levels (*p*<0.0001, both). The levels of TSH and fT4 were inversely associated with age (*p* = 0.0001 and *p* = 0.004, respectively). The level of DHEA-S, however, was markedly correlated with age and level of uric acid (*p*<0.0001, both). Moreover, according to the multiple linear regression analysis, the levels of uric acid and DHEA-S were significantly correlated with age (*p* = 0.0002 and *p*<0.0001, respectively), while the levels of TSH and fT4 were negatively associated with age (*p* = 0.0001 and *p* = 0.0049, respectively) (Table 4).

## Discussion

The level of uric acid, which is low during the neonatal period, increases with age during infancy.^(31)^ In childhood, until the age of 12, uric acid levels are stable and are approximately equal between boys and girls. During early adolescence, however, the levels of uric acid in boys are increased, in contrast to that in girls; this trend persists in adulthood. The sex difference in the level of uric acid among children and early adolescents appears at the prepubertal stage.^(32,29)^ Among obese children and early adolescents, the level of uric acid in obese boys is markedly higher than that in obese girls at 12 years or older.^(30)^ Clinical studies on hormone replacement therapy for transsexual people have demonstrated that sex hormones can affect the level of serum uric acid.^(33,34)^ In rodent experiments, testosterone and estrogen alter the expression of uric acid transporters in renal tubes and affect the uric acid status.^(35,36)^ Thus, uric acid metabolism may be affected by sex hormones too. The present study revealed that the level of serum uric acid in boys with Down syndrome was significantly higher than that in girls. The age at which sex differences in the level of uric acid appear in children with Down syndrome seems to be earlier than in obese or healthy children. However, it is unlikely that boys with Down syndrome are precocious compared to healthy boys. Thus, further research is warranted to ascertain the reason for this observation.

Kubota *et al.*^(29)^ assessed the normal range of uric acid levels, by age, in Japanese children and early adolescents. The cut-off levels of uric acid were 5.9 and 6.1 mg/dl for children aged 7–9 years and 10–12 years, respectively; the cut-off levels of uric acid for children aged 13–15 years were 7.0 and 6.2 mg/dl for boys and girls, respectively.^(29)^ We formulated the cut-off points for uric acid in children with metabolic syndrome using a Receiver Operating Characteristic analysis, which were 4.95 and 5.95 mg/dl for boys (aged <11 years and ≥11 years, respectively) and 4.85 and 5.45 mg/dl for girls (aged <11 years and ≥11 years, respectively).^(30)^ Therefore, in the current study, we defined hyperuricemia in children as serum uric acid levels ˃6.0 mg/dl. Several previous clinical investigations have reported a higher prevalence of hyperuricemia in children and adults with Down syndrome than in a normal cohort.^(37–39)^ The incidence rate of hyperuricemia in Japanese children with Down syndrome is approximately 33%, which is significantly higher than that in healthy children.^(37)^ In the current study, the prevalence rate of hyperuricemia in boys and girls with Down syndrome was approximately 32 and 10%, respectively, which was higher than that of controls reported in previous studies.^(38)^ The current findings corroborated with findings from previous reports.^(37)^ The sex difference evident in the prevalence of hyperuricemia could be attributed to variations in the status of sex hormones as mentioned above.

Kashima *et al.*^(37)^ reported that there was no difference in anthropometric parameters and insulin resistance among Japanese children and early adolescents with Down syndrome, with and without hyperuricemia. Moreover, in the current study, the level of uric acid in children and early adolescents with Down syndrome was associated with age alone, and was not related to other parameters, including anthropometry, thyroid function, non-HDL-C and DHEA-S. Several investigations have demonstrated that uric acid levels in children and early adolescents who are obese are markedly associated with blood pressure, lipids, insulin resistance and anthropometric parameters.^(30,35,40)^ It was proposed that the level of uric acid could be a predictive factor for unhealthy obesity with metabolic and cardiovascular risk in early adolescents and adults.^(41)^ Although it is likely that hyperuricemia in Down syndrome has a high degree of independence from other parameters, confirmation of this hypothesis warrants further research.

Although the exact mechanism of hyperuricemia in children with Down syndrome remains to be determined, there is evidence of increased catabolism of purine and decreased excretion of uric acid.^(42,43)^ Among children and early adolescents with Down syndrome, the intake of purine bodies did not differ between patients with and without hyperuricemia.^(37)^ Throughout lifetime, a patient with Down syndrome is exposed to high levels of oxidative stress, which in turn leads to the overexpression of several genes on chromosome 21.^(44)^ Moreover, oxidative stress affects development and aging in Down syndrome. Uric acid is known to have antioxidant activity, and thus, may compensate for ceaseless oxidative stress.^(45)^ However, the state of oxidative stress in patients with Down syndrome might in turn be related to the metabolism of uric acid. Thus, it has been proposed that the level of serum uric acid should be measured from early childhood in patients with Down syndrome to prevent the development of lifestyle-related diseases in adulthood.^(37)^

Subclinical hypothyroidism is defined as TSH levels above the upper limit of the reference range, with normal levels of fT4 and without clinical manifestations of hypothyroidism.^(46)^ The prevalence of subclinical hypothyroidism in the adult population, and in children and adolescents (aged 0.5–16 years) is 4–20% and 2.9%, respectively.^(46,47)^ There were no sex differences in the prevalence of mild elevated TSH levels (5.5–10 mIU/L; 3.1 and 2.7% for boys and girls, respectively) in a large cohort study (with 12,000 children aged 0.5–16 years).^(47)^ Subclinical hypothyroidism in children with Down syndrome is an abundantly common occurrence with a prevalence of approximately 30%,^(48)^ which has been attributed to the dysregulation of the hypothalamic-pituitary-thyroid axis.^(49)^ However, the use of levothyroxine treatment for children with subclinical hypothyroidism is currently controversial.^(48)^ Alternatively, Down syndrome also has a higher incidence of autoimmune diseases, which enhances the risk of developing autoimmune thyroid diseases.^(49)^ The prevalence of thyroid antibodies in individuals with Down syndrome is 13–34% and increases with age.^(49)^ In the present study, the prevalence of subclinical hypothyroidism among both boys and girls with Down syndrome was higher than that in the general population, with no sex differences, and corroborated with that of the pediatric population.^(47)^ Thyroid antibodies were detected in 13% of the enrolled participants in the current study, emphasizing the need to follow their thyroid function more closely.

Many investigators around the world have reported reference intervals for thyroid hormones in the pediatric population. From birth through adolescence, the levels of thyroid hormones, including TSH, fT3 and fT4 declined with age.^(50–55)^ The age-dependent reduction in thyroid hormones suggests a decline in the demand for thyroid hormones with age during childhood.^(50)^ Conversely, some studies report conflicting findings with respect to a sex difference in thyroid hormone levels during childhood. There was no sex difference in thyroid hormones, including TSH, fT3 and fT4, in the clinical studies on Italian and German children.^(51,52)^ Kapelari *et al.*^(53)^ demonstrated a higher level of fT3 in boys than in girls in an Australian cohort. Further, although the level of fT4 was higher in boys (aged 15–17 years) than in girls of the same age, in a Canadian population,^(54)^ the level of fT4 was lower in boys in a cohort of children from the United States.^(50)^ Among Indian children and adolescents, the levels of fT3 and fT4 were higher in boys than in girls.^(55)^ Moreover, in the current study, the levels of TSH and fT4 in children and early adolescents with Down syndrome were negatively associated with age, which was in line with findings from previous reports.^(50–55)^ Thus, sex differences in the levels of thyroid hormones varied by ethnicity and region. In the present study, however, we excluded children and early adolescents with Down syndrome who were receiving thyroid medication, which may have affected the sex-related results of thyroid hormones.

Obesity is commonly observed in individuals with Down syndrome throughout childhood and into adulthood,^(56)^ and can be attributed to a lower resting metabolic rate in this population.^(57)^ Obesity in children and adolescents is a risk factor for obstructive sleep apnea.^(3)^ Obesity in childhood is carried on into adulthood, which is then a further risk for the development of metabolic syndrome.^(26)^ The incidence of metabolic syndrome in adults with Down syndrome is approximately 21%.^(58)^ In the current study, the prevalence of obesity and being overweight in boys and girls with Down syndrome was approximately 16 and 13%, respectively, which was relatively similar or slightly higher compared than that in the general pediatric population in Japan (i.e., approximately 15 and 11%, respectively).^(59)^ However, since the sample size in the present study is small, prompt evaluation of the prevalence of obesity among children with Down syndrome is challenging. Although patients with Down syndrome visit clinics regularly for medical checks during childhood and early adolescence, they are predisposed to receive incompatible primary care as adults.^(3,60)^ Therefore, ongoing adequate healthcare from young adult stage through older adulthood is crucial to prevent the development of obesity and increased risk of cardiovascular diseases in these patients.

DHEA and its sulfate ester, DHEA-S, are secreted from the adrenal cortex, and converted to sex steroids, including androgens and estrogens, which act on peripheral target tissues.^(61)^ The levels of DHEA and DHEA-S vary throughout the lifetime of a human. It increases with age, until early adulthood, and then declines throughout adult life. There are diverse findings with respect to the pubertal development of patients with Down syndrome. Angelopoulou *et al.*^(62)^ reported that the profiles of hormones, including DHEA-S, estradiol, and follicle stimulating hormone (FSH), in women with Down syndrome in their twenties were not altered compared with control participants. The average age of menarche among Danish girls with Down syndrome was similar to that of controls,^(63)^ whereas Japanese girls with Down syndrome had earlier menarche compared with the general population.^(64)^ Concerning the pubertal development in male patients with Down syndrome, Arnell *et al.*^(65)^ demonstrated primary gonadal insufficiency in this patient population. The levels of other hormones, such as FSH, luteinizing hormone and testosterone, however, were similar between male adolescents with and without Down syndrome.^(66)^ In the current study, the level of DHEA-S was significantly correlated with age, which corroborated with findings in a healthy population. However, the ages of the enrolled participants in the current study only ranged from 6 to 15 years; therefore, a large-scale investigation is required to precisely assess pubertal development in patients with Down syndrome.

Several limitations in the current study must be noted. First, this single center, comparative cross-sectional study had a small sample size, and thus could not sufficiently evaluate the association between several parameters in patients with Down syndrome. Second, we did not account for the pubertal stage of the enrolled participants. Third, we did not assess blood pressure, waist circumference, and fasting glucose levels, which are components of the criteria for the diagnosis of metabolic syndrome.^(67)^ Finally, we only measured anti-thyroid antibodies in a proportion of enrolled individuals with Down syndrome.

In conclusion, we presented the association among biochemical data, thyroid function, and anthropometric parameters in Japanese children and early adolescents with Down syndrome. The prevalence of obesity and being overweight did not significantly differ between boys and girls. The level of uric acid was higher in boys than in girls. Moreover, the prevalence of hyperuricemia was also higher in boys than in girls (approximately 32 and 10%, respectively). The prevalence of subclinical hypothyroidism in children with Down syndrome was approximately 20%, and did not significantly differ between boys and girls. The levels of uric acid and DHEA-S were positively associated with age, whereas the levels of TSH and fT4 were negatively related to age. Because a higher incidence of hyperuricemia is seen during childhood in patients with Down syndrome, the uric acid level and thyroid function must be carefully monitored from childhood through early adulthood to prevent increased cardiovascular risk. Further large-scale, longitudinal investigations are required to validate the findings in the current study on children with Down syndrome.

## Figures and Tables

**Table 1:** Characteristics of all boys and girls with Down syndrome

	Total (*n* = 102)	Boys (*n* = 62)	Girls (*n* = 40)	*p* value
Age (years)	9.6 ± 2.7	9.2 ± 2.8	10.1 ± 2.5	0.0892
**Physical Data**				
Height (cm)	124.6 ± 15.8	124.5 ± 17.8	125.9 ± 13.6	0.6637
Body Weight (kg)	28.9 ± 10.8	28.7 ± 11.8	29.5 ± 10.2	0.7182
Height SD	−2.1 ± 1.1	−1.9 ± 1.1	−2.3 ± 0.9	0.1079
BMI-p	55.5 ± 25.6	57.7 ± 25.3	52.0 ± 26.3	0.257
**Biochemical Data**				
ALT (IU/L)	20.8 ± 11.2	20.8 ± 9.7	19.8 ± 12.5	0.7155
UA (mg/dl)	5.2 ± 1.2	5.5 ± 1.2	4.8 ± 1.0	0.0034*
Non HDL-C (mg/dl)	108.9 ± 24.1	109.1 ± 25.3	108.3 ± 21.9	0.8924
DHEA-S (ng/dl)	97.2 ± 67.5	105.9 ± 76.3	82.1 ± 45.4	0.0952
TSH (µU/ml)	3.7 ± 2.0	3.7 ± 2.1	3.5 ± 1.8	0.6224
fT3 (pg/ml)	3.9 ± 0.6	4.0 ± 0.4	3.8 ± 0.7	0.1725
fT4 (ng/dl)	1.3 ± 0.4	1.3 ± 0.2	1.2 ± 0.2	0.0344

All data are expressed as the mean ± SD. BMI-p, body mass index-percentile; ALT, alanine aminotransferase; UA, uric acid; non HDL-C, non high-density lipoprotein cholesterol; DHEA-S, dehydroepiandrosterone-sulfate; TSH, thyroid-stimulating hormone; fT3, free triiodothyronine; fT4, free thyroxine.

**Table 2:** Number of subjects with several complications among children with Down syndrome

Complications (Number: boys/girls)	Total	Boys	Girls	*p* value
	Number (%)			
Obesity + Overweight (62/40)	15 (14.7)	10 (16.1)	5 (12.5)	0.613
Obesity	3 (2.9)	2 (3.2)	1 (2.5)	
Overweight	12 (11.8)	8 (12.9)	4 (10.0)	
Hyperuricemia (62/40)	24 (23.5)	20 (32.3)	4 (10.0)	0.0097*
Subclinical hypothyroidism (62/40)	20 (19.6)	11 (17.7)	9 (22.5)	0.555
Positive of thyroid antibodies (55/36)	12 (13.2)	3 (5.5)	9 (25.0)	0.007*
TSAb	5 (5.5)	1 (1.8)	4 (11.1)	
TRAb	7 (7.7)	1 (1.8)	6 (16.7)	
TPOAb	8 (8.8)	1 (1.8)	4 (11.1)	

TSAb, thyroid-stimulating antibody; TRAb, thyroid-stimulating hormone receptor antibody; TPOAb, antithyroid peroxidase antibody.

**Table 3:** Correlation coefficient among several parameters in individuals with Down syndrome

Dependent variables	Independent Variables [r^2^ (*p* value)]					
	Age	UA	non HDL-C	TSH	fT4	DHEA-S
UA	0.21 (<0.0001)	—	0.02 (0.2090)	0.01 (0.2918)	0.00 (0.6159)	0.20 (<0.0001)
Non HDL-C	0.01 (0.3421)	0.02 (0.2090)	—	0.01 (0.2759)	0.00 (0.8577)	0.00 (0.734)
TSH	0.14 (0.0001)	0.01 (0.2918)	0.01 (0.2759)	—	0.00 (0.8929)	0.02 (0.1737)
fT4	0.08 (0.004)	0.00 (0.6159)	0.00 (0.8577)	0.00 (0.8929)	—	0.00 (0.9564)
DHEA-S	0.34 (<0.0001)	0.20 (<0.0001)	0.00 (0.734)	0.02 (0.1737)	0.00 (0.9564)	—

Abbreviations are listed in Table 1.

**Table 4:** Significant of determinants of uric acid, TSH and DHEA-S with variable parameters in multiple regression analysis

Dependent variables	Independent variables	b	95%CI	*p* value	b’
UA	Age	0.2166544	0.1064364 to 0.3268724	0.0002	0.480933
DHEA-S	Age	15.799602	10.626847 to 20.972358	<0.0001	0.647262
TSH	Age	−0.413795	−0.620437 to −0.207153	0.0001	−0.55851
fT4	Age	−0.035277	−0.059574 to −0.010981	0.0049	−0.43371

Abbreviations are listed in Table 1, and analysis is adjusted by sex.

## Abbreviations

ALT: alanine aminotransferase
BMI-p: body mass index-percentile
DEHA: dehydroepiandrosterone
DHEA-S: dehydroepiandrosterone-sulfate
ECLIA: electrochemiluminescence immunoassay
FSH: follicle stimulating hormone
fT3: free triiodothyronine
fT4: free thyroxine
HDL-C: high-density lipoprotein cholesterol
TPOAb: anti-thyroid peroxidase antibody
TRAb: thyroid-stimulating hormone receptor antibody
TSAb: thyroid-stimulating antibody
TSH: thyroid-stimulating hormone
UA: uric acid
